# Easier said than done!: methodological challenges with conducting maternal death review research in Malawi

**DOI:** 10.1186/1471-2288-14-29

**Published:** 2014-02-21

**Authors:** Viva Combs Thorsen, Johanne Sundby, Tarek Meguid, Address Malata

**Affiliations:** 1 Department of Community Medicine, University of Oslo, PO Box 1130, Blindern, N-0318 Oslo, Norway; 2 Department of Obstetrics & Gynaecology, University of Namibia, School of Medicine, Private Bag 13301, Windhoek, Namibia; 3 Department of Maternal and Child Health, Kamuzu College of Nursing, University of Malawi, Private Bag 1, Lilongwe, Malawi

**Keywords:** Maternal death review, Maternal death audit, Maternal mortality, Surveillance cycle, Malawi

## Abstract

**Background:**

Maternal death auditing is widely used to ascertain in-depth information on the clinical, social, cultural, and other contributing factors that result in a maternal death. As the 2015 deadline for Millennium Development Goal 5 of reducing maternal mortality by three quarters between 1990 and 2015 draws near, this information becomes even more critical for informing intensified maternal mortality reduction strategies. Studies using maternal death audit methodologies are widely available, but few discuss the challenges in their implementation. The purpose of this paper is to discuss the methodological issues that arose while conducting maternal death review research in Lilongwe, Malawi.

**Methods:**

Critical reflections were based on a recently conducted maternal mortality study in Lilongwe, Malawi in which a facility-based maternal death review approach was used. The five-step maternal mortality surveillance cycle provided the framework for discussion. The steps included: 1) identification of cases, 2) data collection, 3) data analysis, 4) recommendations, and 5) evaluation.

**Results:**

Challenges experienced were related to the first three steps of the surveillance cycle. They included: 1) identification of cases: conflicting maternal death numbers, and missing medical charts, 2) data collection: poor record keeping, poor quality of documentation, difficulties in identifying and locating appropriate healthcare workers for interviews, the potential introduction of bias through the use of an interpreter, and difficulties with locating family and community members and recall bias; and 3) data analysis: determining the causes of death and clinical diagnoses.

**Conclusion:**

Conducting facility-based maternal death reviews for the purpose of research has several challenges. This paper illustrated that performing such an activity, particularly the data collection phase, was not as easy as conveyed in international guidelines and in published studies. However, these challenges are not insurmountable. If they are anticipated and proper steps are taken in advance, they can be avoided or their effects minimized.

## Background

The World Health Organization (WHO) estimated that 287 000 women died worldwide due to pregnancy-related complications in 2010 [1]. Resource-poor countries accounted for 99% of these deaths, with Sub-Saharan Africa disproportionately representing nearly three fifths of these deaths (162 000). The tragedy is that the majority of these deaths are avoidable when adequate care is given and preventive measures taken. To address maternal mortality, effective preventive measures must be based on correct information. Clinical and epidemiological information alone are insubstantial for preventing further deaths. In-depth information about why women are dying is required [2]. One approach to obtaining this information is to systematically review all maternal deaths for a specified period of time [3,4].

The WHO developed a handbook to assist in conducting such reviews, “Beyond the numbers: Reviewing maternal deaths and complications to make pregnancy safer” [5]. For resource-poor settings, confidential enquiries, and community- and facility-based reviews are easiest to promote and implement because they do not require external expertise, advanced academic rigor or sophisticated questionnaires [6]. In particular, the facility-based maternal death review approach is one of the oldest and extensively documented methods in resource-poor settings [7-12]. Nonetheless, implementation of this approach is associated with difficulties.

In Malawi, where maternal death reviews have been institutionalized at the district level, two related studies reported on challenges in implementing the maternal death review process. The first study was cross-sectional [13]. The aim of this study was to identify the causes and characteristics of 43 maternal deaths that occurred in nine hospitals in the central region of Malawi between January and December 2007. Another aim of this study was to identify problems encountered during the review process. Kongnyuy and colleagues reported that the maternal death review committees faced several problems that included a shortage of senior staff to conduct the reviews, partial confidentiality and anonymity, poor documentation and record keeping, and a lack of resources and commitment to implement the recommendations.

The second study, which was exploratory in nature, was conducted by Kongnyuy and van den Broek [4]. The purpose of this study was to identify push and pull factors involved in implementing the maternal death review process. They used the strengths, weaknesses, opportunities and threats analysis framework during a one-day workshop with 60 Malawian health professionals who were from the same nine hospitals in the central region that were included in the first study. Kongnyuy and van den Broek noted that although Malawi’s maternal death review process was supported by district health management teams and standardized maternal death review forms were available, a fear of blame and poor record keeping impeded its successful implementation. A lack of knowledge and skills for the proper conducting of reviews was also mentioned. The participants generated strategies to exploit the strengths and opportunities, and identified strategies to overcome the weaknesses and threats. Those strategies included: promoting community involvement, ensuring anonymity and confidentiality during maternal death reviews, encouraging districts to allocate resources for the implementation of recommendations from maternal death reviews and lobbying for more staff from the Ministry of Health.

More recently, van Hamersveld and colleagues [14] explored barriers to implementing an obstetric audit at Saint Francis Designated District Hospital in Ifakara, Tanzania. They conducted participant observations and in-depth interviews with 23 healthcare workers who reported that audit sessions were only convened when the head of the department of obstetrics and gynecology was available. They also stated that insufficient staff commitment, managerial support and resources all contributed to poor implementation of recommendations that resulted from the audit session. Moreover, van Hamersveld and colleagues observed that participants had inadequate knowledge of the purpose of the audits [14]. These studies focused on the entirety of the maternal death review process, with a heavy emphasis on establishing a committee and implementing the recommendations.

Because the district health management teams throughout Malawi were overloaded with maternal death cases to review, the Ministry of Health encouraged all hospitals to individually review maternal deaths that occur at their respective facilities [4]. For some of those who are responsible for carrying out this activity, it will be a daunting enigmatic task that will require decoding. By systematically highlighting the challenges, the maternal death review process itself may become understandable.

The aim of this paper is to critically reflect upon the process used to carry out a facility-based maternal death review study, while highlighting challenges and providing recommendations on how best to overcome these challenges. Our findings are expected to inform members of maternal death review committees or researchers aiming to engage in similar studies, as well as policy makers prioritizing funds, maternal healthcare personnel modifying their care practices and district safe motherhood programs fulfilling their responsibilities.

## Methods

In this paper, we critically reflected upon the five-step maternal death surveillance cycle that was used to carry out a facility-based maternal death review study in Lilongwe, Malawi. That study was carried out in compliance with the Helsinki Declaration. It was approved by The College of Medicine Research Ethics Committee in Malawi (Proposal No. 10/08/703) and The Regional Committee for Medical and Health Research Ethics in South-Eastern Norway (2008/16105). In brief, that study was primarily conducted in an urban setting at comprehensive emergency obstetric care units at a secondary hospital and a tertiary hospital. Women who died during the period from January 1, 2011 to June 30, 2011 while pregnant or within six weeks of termination of pregnancy and had resided in the Lilongwe district area prior to death were included in the study. Their charts (n = 32) were reviewed and important information such as parity, gravidity, medical history, antenatal visits, and differential diagnosis was extracted using a medical record extraction form. Healthcare workers (n = 34) who provided care to the deceased women in question were interviewed using the Facility Staff Interview Questionnaire which included open-ended and semi-structured questions to document healthcare workers’ accounts of the death in question, the actions taken, the challenges with providing care, and avoidable factors. With the aid of the Verbal Autopsy and Contributing Factors Questionnaire, family members (n = 27), including guardians and traditional birth attendants, were interviewed. The questionnaire included five sections: 1) background, 2) symptoms; 3) existing diseases 4) health seeking behavior/ contributing factors and 5) family’s account of events surrounding the woman’s death and illness. All three data collection tools were adapted from the WHO guidelines: “Beyond the numbers reviewing maternal deaths and complications to make pregnancy safer” [5].

Data were collected by the first author and two research assistants whose characteristics are summarized in Table 1. The first author explained the purpose and objectives of the study to each of the research assistants. The roles, duties and what was expected of the research assistants were also explained. The first author then guided them through each data collection form and gave them the opportunity to voice comments, questions and concerns. The chart extraction form was piloted using two maternal death cases from the previous year. However, the questionnaires were not piloted because it was believed that both assistants had adequate interviewing skills and that a significant amount of time would have been expended tracking the appropriate personnel and families.

**Table 1:** Characteristics of the researcher, assistants, and participants

	**Researcher**	**Research assistant (I)**	**Research assistant (II)**	**Participants (Healthcare workers)**	**Participants (Families)**
**Job title**	**Research fellow**	**Nurse Midwife Technician (NMT)**	**Research nurse**	**NMTs, clinical officers, matrons, RNs, NM**	**Unemployed, farmers, small business owners, and a teacher**
Gender	Female	Female	Male	Mixed	Mixed
Age	40	39	33	Range: 24 - 47	Range: 23 – 60+
Marital status	Married	Married	Married	Mixed	Mixed
No. of children	2	2	2	?	?
No. of years of education	20+	15	15	Range: 15-17	Range: 3-16
Economic status	Non-poor	Non-poor	Non-poor	Non-poor	Mostly Poor
Religious affiliation	Christian (Baptist/Lutheran)	Christian (Catholic)	Muslim	Mixed	Mixed
Nationality	American (African descent)	Malawian	Malawian	Malawian + 1 Burundian	Malawian
Research experience	Some experience working with research assts	Some experience as research asst/interpreter	Very experienced as research asst/interpreter	Range of experience as research participants	No experience participating in research

The transcripts from both questionnaires were analyzed using a directed approach to the content analysis [15]. The data from the respective medical chart and interview transcripts were triangulated to obtain a more accurate account of what transpired. Based on the International Classification of Diseases 10th version (ICD-10), two obstetricians/gynecologists (OB/GYNs) independently reviewed the triangulated data for each maternal death and determined the causes of death. They confirmed the documented causes of deaths or provided alternative causes. One of the OB/GYNs who was instrumental in establishing the Norwegian-Physician Exchange Program (Norwegian doctors provided technical assistance, worked in the maternity ward for a 6-month rotation) has worked at the study sites since 2005. The other OB/GYN has over 20 years of experience working in various countries in Africa, including Malawi. Detailed descriptions of the methodology and the research findings are published elsewhere [16].

The critical reflection process was approached by asking “what happened?”, “what went well?”, “what did not go well?”, “why did or did not something happen?”, and “how this might be done differently in the future?” Field and journal notes, along with notes from debriefing sessions with research assistants, were reviewed in light of these questions. The data collection process, results of data analyses and interpretations were compared with those of published maternal mortality studies and other relevant literature. These activities helped gain new understanding, perspectives and appreciation for the maternal death audit approach.

## Results

Critical reflection was organized according to the five-step maternal death surveillance cycle used to carry out the facility-based maternal death review as follows: 1) identification of maternal death cases, 2) data collection, 3) reviewing cases and analyzing the data, 4) recommendations, and 5) evaluation. The challenges and the remedial actions taken will now be discussed.

### Identification of maternal death cases

We used the standard WHO definition of maternal death, which is the death of a woman while pregnant or within 42 days of termination of pregnancy, irrespective of the duration and site of the pregnancy, from any cause related to or aggravated by the pregnancy or its management but not from accidental or incidental causes [17]. Our main sources for identifying the maternal death cases were registries in the high-risk antenatal wards, labor wards, post labor wards, and operating theaters. In addition to these sources, we relied heavily on the maternal death report book, the matron registry book and an excel spreadsheet that was maintained by the matrons. These latter three sources were presumed to account for maternal deaths within the hospital, not just the maternity unit’s cases. The number of maternal death cases varied, ranging from nine to 12 for the secondary site and from 22 to 33 for the tertiary site. These discrepancies were brought to the matrons’ attention and they advised the data extractors to use the matron registry book, stating that it was the most up-to-date recording book available. The number of maternal deaths recorded during January 1 to June 30, 2011 was 58, and the number of live births for the same period was 8108. This translated into an institutional maternal mortality ratio of 715 per 100 000 live births. Thirty-nine of the 58 cases had lived in Lilongwe District, and thus were included, while the other 19 had resided outside of Lilongwe District and were not reviewed any further. Seven medical charts were missing. Therefore, a total of 32 maternal death cases were reviewed.

### Data collection

Data collection involved three activities: chart review/extraction, facility-based interviews, and community-based interviews. These activities had their own challenges and are discussed below.

### Chart review/extraction

The first author and the two research assistants extracted information from the medical charts. The challenge with extracting the information was that some of the charts were missing. Seven out of 39 medical charts were not found. The first author and the research assistants looked for the charts at the referring facilities, in an office where they were previously stored and at a non-profit organization’s office that was reviewing charts for quality assurance purposes. They also sought the assistance of the matrons in charge who inquired at other departments, including the clerk’s department, but no one could locate them.

In other instances information was not written or was written but incomplete. Another problem was that entries within the record were illegible or written in phrases and abbreviations. For patients who stayed for more than three to four days, the charts were filled with volumes of information with no standard note taking. There were at least two sets of notes, including those of the nurse midwives and those of the clinician; and sometimes an enrolled nurse wrote notes. Some of the notes were written on pieces of paper, some pages were not inserted chronologically, and some entries did not include the date, time, or signature of who actually performed the task. If a variable was missing the data extractors then reviewed the entire chart a second and third time. If the information was not in the chart then the data extractors searched through the supporting documents. They frequently asked each other what was written and if they could not determine what it said they would then ask the matrons in charge. This activity became tedious, time-consuming, and sometimes unfruitful. The first author regularly met with the two assistants to assess the progress and quality of their extractions, as well as to double check her own entries.

#### Facility-based interviews

Interviews with selected health professionals who provided care to the deceased posed a challenge from the outset. This challenge involved determining whom to interview, particularly when more than one staff member and hospital unit were involved in the care. Interviewing all of the health care staff was not feasible or appropriate, especially when the patient was transferred shortly after admission. Eligible participants were identified by their signatures in the medical charts. In instances where signatures were illegible, or when entries did not have an accompanying signature, the matrons in charge, the healthcare workers themselves or the schedule rosters helped identify the healthcare workers in question. Once the healthcare workers were identified, we needed to locate them, and inform them about the study. Written consent was obtained and they were interviewed.

Locating the healthcare workers was slightly difficult because they had either moved out of town or were on leave during the time of the data collection. Each healthcare worker was given the medical chart to review to help reduce recall bias and to reacquaint him or herself with the case. However, because the study was not part of the hospitals’ formal quality assurance activities, participation was given a low priority.

Some of the questions in the questionnaire itself were problematic. For instance, some questions asked about the participants’ knowledge of the purpose of antenatal visits (e.g. routines or problems), the women’s health status prior to becoming pregnant, and factors affecting the women’s condition prior to arrival. Antenatal visits and health status are typically recorded in a health passport which is kept by the woman herself. Therefore, unless the healthcare worker has access to this passport, she or he is unlikely to answer these questions. Similarly, unless questions regarding events occurring prior to arrival are asked and recorded during admission, the participating healthcare worker will not know this information either. Furthermore, some of the questions related to avoidable factors appeared to lead participants to assume that something took place in the community, blaming the women (e.g., coming late/transportation issues) without any concrete evidence.

Lastly, in two of the cases where the circumstances surrounding the deaths were controversial, some participants actually omitted important information, or gave misleading information. This situation posed a dilemma for us because we had to decide whether to continue to investigate until the true accounts of the event were revealed or to respect the participants’ rights to decide what information to divulge. In these instances more than one healthcare worker was interviewed to ensure a more accurate picture of the events. By emphasizing and repeating the fact that the guiding principle and sole purpose of the maternal death review were to help save lives in the future and not to provide a basis for litigation, management sanctions or to apportion blame participation was ensured.

#### Community-based interviews

The WHO suggests that when possible, facility-based maternal death reviews should also be concerned with identifying the combination of factors that contributed to the death, both at the facility and in the community [5]. Therefore, the maternal deaths reviewed were traced to their respective communities and family and community members were interviewed.

### Family members

From the outset, a major hurdle was identifying where the women lived. During admission to the facility, patients are presumably asked for a home address. In most cases, only the area where the women lived (no sign posts or locators) was recorded. In a few cases a land mark was given, and in even fewer cases the name of the traditional authority was listed. A contact phone number was rarely provided.

Once the eligible participants were identified, obtaining informed consent was met with potential ethical concerns. Firstly, Malawian chiefs are highly esteemed and play a critical role in the consent process. Consequently, prior to inviting family members to participate in the study permission needed to be obtained from the chiefs. Written consent was the initial method chosen. However, because of the low literacy rate among Malawians, particularly among participants living in the rural areas, two alternative methods for obtaining consent emerged. These methods were verbal/oral consent and thumb print which were appropriate for the participants.

Another problem occurred when recapping the events because some of the participants could not recall the exact timing of events. The concept of time was difficult for them (none wore/owned a watch), which made it more difficult to construct a timeline of the events preceding death. Furthermore, the distance travelled was difficult to determine because the mode of transport was usually by foot. These two factors also made it difficult to determine the degree to which the woman and family experienced delays from the onset of a complication to reaching the nearest facility, and from receiving care to death. However, because the interviews were triangulated with interviews from the healthcare workers and information extracted from the medical charts, some of the gaps were filled.

Because documenting participants’ responses accurately is of the utmost importance in qualitative health research, particularly during the data analysis phase and for ensuring the trustworthiness of the findings [18], permission was sought to record the interviews. During the interview, some participants were freer at expressing themselves before the recorder was turned on, which signified the official start of the interview, whereas turning it off officially concluded the interview. Moreover, when the recorder was on, they gave adulterated responses.

Another related problem was with the recording device itself. At one point it was inoperable. Therefore, the voice recorders on a mobile phone and laptop computer were used, while the research assistant took detailed notes., there was a lot of background interference that cancelled out some of the voices because most interviews took place outside. In those instances, field and debriefing notes supplemented the transcripts.

Lastly, the whole process of locating the deceased women’s places of residence, and their relatives was physically and mentally exhausting. An example of this issue is that locating just one family could take an entire day, from 7:30 am to 5:30 pm.

### Traditional birth attendants

Even though traditional birth attendants (TBAs) were involved in the care of four of the 32 maternal death cases reviewed, their perspective was critical for obtaining a clearer idea of the circumstances surrounding the deaths. For this reason, they were tracked down and invited to participate in the study. One of the research assistants and a health surveillance assistant advised the first author not to take notes because the TBAs might not speak candidly about the deaths for fear of being reported to the health authorities^a^. Based on fear or skepticism about our motives, two of the TBAs were not as forthcoming about the degrees to which they assisted the women, (e.g. whether they delivered the baby, the placenta, or only gave advice). As a result, we tended to rely on the guardians’ accounts when there was a discrepancy.

All four TBAs in our study were subject to recall bias; they were unable to provide exact times of events and what they provided to their clients. In one case the TBA could not remember the case in question. The TBAs all stated that they provided various services to several patients in a given month, and that patients typically came in the middle of the night.

### Language barrier

Because the first author and the participants did not speak the same language, interviews were conducted with the two research assistants who also served as interpreters which might have added additional layers of bias within the interview process.

Some participants gave long responses when narrating the events surrounding their loved ones’ deaths which made it difficult for the research assistants to completely recall what took place. In those instances, the research assistants made the decision on what to interpret and share with the first author. There was also a risk that elaboration or limitation of follow-up questions might have occurred. Sometimes the research assistants posed a clarifying question to the participants or began interpreting the participants’ responses in mid-sentence, thereby making some participants lose their train of thought. At other times the participant would return to the subject at hand, while others participants would switch to another topic without completely answering the previous question. To help limit these problems, during debriefing sessions the first author would stress the importance of letting the participants express themselves without interruption. When necessary, she would also explain in detail the purpose and meaning of certain follow-up questions.

### Reviewing cases and analyzing data

The primary outcomes of this step were the medical causes of death which were either direct or indirect, and a maternal death epidemiological profile that describe the magnitude and distribution of the problem of maternal death in a specified time period. Non-medical causes of death were also ascertained during this step. The ICD-10 was used to classify the causes of maternal deaths for the 32 cases reviewed.

Because the two independent OB/GYNs did not take part in collecting the data or were not present in Malawi during the data analysis, they did not have direct access to the women’s medical charts, the healthcare workers, or the women’s families. Furthermore, the causes of death were elucidated through proxies, in the forms of triangulated extracted medical chart data, interview transcripts, and their clinical judgment. Because the first author does not have a medical background, follow-up questions posed by the reviewers could not be adequately answered. Furthermore, the analysis took place in Norway, which made reaching important Malawian personnel difficult. Through the process of classifying the causes of deaths, the independent reviewers were found to be in agreement with the facilities’ reported causes of death in only seven of the 32 cases (Table 2).

**Table 2:** Facility and independent reviewers’ cause of death classifications

**Case**	**Facility classification**	**Independent reviewers**	**ICD-10 code and comments**
	**Cause of death**		
**01**	Cardiac arrest, Malaria & Anemia	Disagree	O99.0; O98.6; O99.4-I50.9 Protozoal diseases (Malaria) complicating pregnancy, childbirth and the puerperium; Anemia complicating pregnancy, childbirth and the puerperium; Diseases of the circulatory system complicating pregnancy, childbirth and the puerperium – Heart failure unspecified.
			*Malaria is the cause of the woman’s severe anemia. Her cardiac arrest is her mode of death.*
**02**	Valvular heart disease	Disagree	O99.4-I50.9 (O13 cofactor) Diseases of the circulatory system complicating pregnancy, childbirth and the puerperium – Heart failure unspecified (Gestational [pregnancy-induced] hypertension without significant proteinuria).
			*Valvular heart disease is a very likely cause of heart failure in settings where late complications following rheumatic fever in childhood are commonplace. However, I simply question the validity of this diagnosis in this specific case. The findings available in the notes demonstrate high blood pressure and heart failure. The cardiac lesion may have been congenital. This is an example of where a common cause is inferred but not confirmed, but believed because, as it is said in medicine, “common is common”.*
**03**	Puerperal sepsis	Agree	O85 Puerperal Sepsis
**04**	Peritonitis postpartum	Disagree	O85; O73.1 Puerperal sepsis; Retained portions of placenta and membranes, without hemorrhage
			*The woman has a form of puerperal sepsis as is suggested by the clinicians. In the notes it seems evident that a contributing factor to the development of this disease process was the presence of retained products of conception.*
**05**	Pre-eclampsia	Disagree	O85; O73.1 Obstetric blood-clot embolism; Pulmonary edema; Severe pre-eclampsia
			*The woman develops severe respiratory distress and dies. She has pre-eclampsia. Two causes are possible that can arise as a result of pre-eclampsia. The first is a thrombotic pulmonary embolism. The second would be severe pulmonary edema. Both of these conditions may give rise to her clinical presentation. An autopsy would be able to discern between the two possible causes.*
**06**	Renal failure due to puerperal sepsis	Disagree	O90.4; O85 Postpartum acute renal failure: Puerperal sepsis.
			*The woman developed renal failure following delivery. She also developed puerperal sepsis. The renal failure is likely to be secondary to the development of sepsis, but the two conditions may be coincidental. The renal failure may have a separate, unknown cause. The mode of death is a failure of the woman’s kidneys to function. The cause is likely to be septicemia.*
**07**	Lactic acidosis (HIV-related)	Disagree	O98.7; B20.9 HIV disease complicating pregnancy, childbirth and the puerperium; HIV disease resulting in unspecified infectious or parasitic disease.
			*Lactic acidosis is a physiological state leading to, but not a cause of, death. The woman was sick and was HIV positive. I am inferring that the patient died of complications arising from AIDS.*
**08**	Cryptococcal meningitis	Disagree	O98.8; B45.1; O98.7 Other maternal infectious and parasitic diseases complicating pregnancy, childbirth and the puerperium; Cerebral cryptococcosis; HIV disease complicating pregnancy, childbirth and the puerperium.
			*Cryptococcal meningitis is a very serious and common cause of meningitis in people who are HIV positive. However, it must be noted that even though the woman was meningitic and HIV positive, the diagnosis of Cryptococcus is not confirmed by lumbar puncture. The causative organism may have been one other than Cryptococcus.*
**09**	Heart failure pulmonary embolism	Disagree	O88.2; O99.4-I50.9 Obstetric blood-clot embolism; Diseases of the circulatory system complicating pregnancy, childbirth and the puerperium – Heart failure unspecified
			*The most likely predisposing factor for an otherwise fit and healthy woman who develops a pulmonary embolism is thrombosis secondary to pregnancy.*
**10**	Hypoxia, eclampsia	Disagree	O15 Eclampsia inn pregnancy.
			*The cause of this woman’s death was eclamptic seizures. Her mode of death was lack of oxygen (hypoxia).*
**11**	Concealed ruptured uterus due to obstructed labor	Disagree	O66.9 Obstructed labor, unspecified.
			*Ruptured uterus is a complication of obstructed labor in a parous uterus. It is not always complicated by profound bleeding. The hemorrhage is the mode. Obstructed labor is the cause.*
**12**	PPH	Agree	O72 Postpartum hemorrhage.
**13**	Intrapartum hemorrhage due to ruptured uterus	Disagree	O67.9; O71.1; O34.2 Intrapartum hemorrhage, unspecified; Rupture of uterus during labor; Maternal care due to uterine scar from previous surgery.
			*For a uterus to rupture in the presence of a previous scar the labor does not necessarily need to be obstructed. If anemia arises as a result of rupture then it is secondary to a hemorrhage that is perhaps a little less profound, but just as lethal as the last case.*
**14**	Valvular heart disease - stenosis	Disagree	O99.4; I50.9 Diseases of the circulatory system complicating pregnancy, childbirth and the puerperium – Heart failure unspecified.
			*As in case no. 2, the cardiac lesion is assumed to be valvular stenosis. I do not doubt this condition as it is not an uncommon cardiac condition in Malawi. It will lead to congestive cardiac failure (more mode than cause). However, I felt that the diagnosis given was lacking confirmation in the details provided and was therefore assumed to be inferred.*
**15**	PPH	Agree	O72 Postpartum hemorrhage.
**16**	Hypovolemic shock	Disagree	O72 Postpartum hemorrhage.
			*Can arise from septicemia, and it can arise from exsanguinations. To me, the details available were most suggestive of shock (mode) secondary to a profound blood loss (cause). PPH*
**17**	Hypoxia and severe pneumonia	Disagree	O99.4-I50.9; O99.0 Diseases of the circulatory system complicating pregnancy, childbirth and the puerperium – Heart failure unspecified; Anemia complicating pregnancy, childbirth and the puerperium.
			*The patient was severely anemic. This will result in high output cardiac failure and respiratory distress. The terminal symptoms can mimic those of a patient presenting with severe pneumonia. However, I was not there. My opinion is based on the details presented.*
**18**	PPH	Agree	O72 Postpartum hemorrhage.
**19**	Bacterial meningitis/severe malaria	Disagree	O98.6; Assumed B50.0 Protozoal diseases (Malaria) complicating pregnancy; Assumed Plasmodium falciparum malaria with cerebral complications. *The woman was in a coma and had malaria. She may have had cerebral malaria. Bacterial meningitis was never confirmed by lumbar puncture.*
**20**	Severe anemia	Disagree	O99.4-I50.9; O99.0 Diseases of the circulatory system complicating pregnancy, childbirth and the puerperium – Heart failure unspecified; Anemia complicating pregnancy, childbirth and the puerperium.
			*Anemia (cause) resulting in circulatory collapse (mode).*
**21**	Severe anemia	Disagree	O72; O99.0 Postpartum hemorrhage; Anemia complicating pregnancy, childbirth and the puerperium.
			*Two causes. Neither were necessarily lethal enough alone, only in combination.*
**22**	Peritonitis postpartum	Disagree	O85; O86.0 Puerperal sepsis; Infection of obstetric surgical wound (C-section).
			*The woman had a puerperal sepsis, however it is important to highlight the fact that she underwent surgery prior to developing overt signs of infection. The surgery may be only coincidental. However, the details of this case suggest that the surgery was contributory to the development of lethal infection.*
**23**	Hepatic failure	Disagree	O90.4 Hepatorenal syndrome following labor and delivery
			*Hepatorenal failure is a specific ICD-10 code. There may be differing etiologies. In the absence of pre-existing liver or renal dysfunction one assumes that the cause of this organ failure was pregnancy-related. An example of this would be Acute fatty liver of pregnancy (AFLP).*
**24**	Eclampsia	Agree	O15.0 Eclampsia in pregnancy.
**25**	Anemia	Agree	O99.0 Anemia complicating pregnancy, childbirth and the puerperium.
**26**	Anemia	Disagree	O99.0; O98.7 Anemia complicating pregnancy, childbirth and the puerperium; HIV disease complicating pregnancy, childbirth and the puerperium *Anemia is a cause. The woman’s constitution was weakened as a result of her HIV infection which therefore contributory to the anemia, thus resulting in death.*
**27**	PPH	Disagree	O72; O45.9 Postpartum hemorrhage; Premature separation of placenta, unspecified.
			*The history sounds typical of a grade 3 abruption of the placenta. If coagulopathy results then this will in turn result in deadly hemorrhage if large transfusion of blood products is not performed immediately.*
**28**	Hypovolemic shock	Disagree	O03.5 Spontaneous abortion - complete or unspecified, complicated by genital tract and pelvic infection.
			*The woman presented with an incomplete miscarriage with signs of infection (cause). This resulted in hypovolemic shock and subsequent death (mode). The question is whether the miscarriage was spontaneous or procured.*
**29**	Septicemia post delivery	Disagree	O85; O98.7; O98.6 Puerperal sepsis; HIV disease complicating pregnancy, childbirth and the puerperium; Protozoal diseases (Malaria) complicating pregnancy.
			*Malaria was the working diagnosis presented in the details of the case, in a woman whose constitution is weakened by HIV. Other sources for infection are not forthcoming in the details, but that does not mean that they were not present.*
**30**	Lactic acidosis (HIV-related)	Disagree	O98.6; O99.0 Protozoal diseases (Malaria) complicating pregnancy; Anemia complicating pregnancy, childbirth and the puerperium.
			*Lactic acidosis is a physiological state that, if profound, can lead to death (mode). The details of the case highlight both malaria and coincidental or subsequent anemia.*
**31**	Sepsis and induced abortion	Agree	O07.8 Failed attempt abortion with other and unspecified complications.
**32**	Cardiac arrest, severe pre-eclampsia	Disagree	Her cardiac arrest was her mode of death. *(Unfortunately for this case, the comments have been lost)*

For each maternal death case, the data from the respective medical chart and interview transcripts were triangulated. Based on the International Classification of Diseases tenth version (ICD-10), clinical judgment and experience, two OB/GYNs independently reviewed the triangulated data for each maternal death and determined the causes of deaths. They either agreed or disagreed with the reported cause of death. If they disagreed they provided an alternative cause(s), along with comments.

### Synthesis of analyzed data

Through the maternal death review process, a large amount of information on factors that contributed to the death was generated. The most widely used framework for analyzing qualitative data in maternal death research is the Three Delays Model. Thaddeus and Maine asserted that in most cases, if prompt, adequate treatment is provided, adverse outcomes can be avoided [19]. They proposed that pregnancy- related mortality overwhelmingly contributes to delays in three phases: (1) deciding to seek appropriate medical help for an obstetric emergency; (2) reaching an appropriate obstetric facility; and (3) receiving adequate care when a facility is reached.

### Recommendations

The fourth step in the maternal death surveillance cycle is to provide recommendations based on what was determined from reviewing cases and analyzing data. The factors (community and facility-based) that contributed to the maternal deaths and the healthcare delivery system in which the deaths occurred, were both complex and intricately woven into all sectors Consequently, the recommendations given were multidisciplinary.

The findings and recommendations were presented to healthcare workers and senior management at the two hospitals where the study took place. A detailed prescription of solutions to address delays in accessing, receiving, and providing quality emergency obstetric care was deliberately not provided. Instead, prioritizing practical solutions for delays in providing care and delays in deciding to seek care was suggested. To help assist in the prioritizing process a birth-preparedness and complication readiness matrix was proposed [20-22]. Further investigations that honed in on skilled attendants were also suggested. Devising interventions based on these future investigations was thought to have an impact on maternal mortality. The findings and recommendations have not been shared with communities yet.

### Evaluation

The fifth step has yet to be conducted. However, actions will hopefully be planned, taken, and evaluated based on the recommendations. By reviewing maternal death cases in the future, and paying particular attention to the problems identified in our study, the cycle then becomes a continuous quality assessment and quality improvement cycle.

## Discussion

Although challenges were faced throughout the research process, they were surmountable. We discuss our findings according to the five-step maternal death surveillance cycle. Strategies to overcome or minimize the potential problems that might be encountered in future facility studies are provided. Implications for improving clinical practices through implementing the five steps are also discussed (Table 3).

**Table 3:** Maternal death surveillance cycle and implications for participating hospitals

**Step in maternal death surveillance cycle**	**Implications for health facility**
1. Identification of cases	Develop policy (or enforce existing policy) that all maternal cases within the hospital be reported to a designated unit (possibly the clerk’s office of the maternity unit?).
	Someone from the maternity unit should be designated to routinely (e.g. weekly or biweekly) visit (or call) all the possible departments where a woman of reproductive age may receive care and inquire about deaths and determine pregnancy status and cross check maternal death case numbers.
2. Data collection	Standard case note taking and medical record maintenance/storage should be enforced. Remedial training and sensitization on the importance of good record keeping practices should be provided. Systematic case-note audits should be regularly conducted. Additional funding from the Ministry of Health and appropriate collaborative partners should be provided to sustain and institutionalize good record keeping practices.
	Coordinate with the safe motherhood program (or designated community health personnel) so they can follow up with cases in the community.
3. Data analysis	Basic and advanced courses/training in ICD-10 coding, cause of death certification should be provided for appropriate healthcare personnel.
	Medical doctor or medical officer should facilitate maternal death audits.
	Feedback loop to all appropriate healthcare staff, especially to those who were involved with caring for the patient in question.
4. Recommendations	Healthcare personnel formulate recommendations with senior staff.
	Additional funding from the Ministry of Health and appropriate collaborative partners should be provided to implement the recommendations.
	Senior staff should take lead and ensure recommendations are implemented.
5. Evaluation	Indicators should be formulated and agreed upon by healthcare personnel and senior management. Routine evaluation conducted accordingly.

### Identification of maternal death cases

Even though all maternal deaths were presumably reported to the maternity unit, some indirectly-caused maternal deaths might have been missed [5] or maternal deaths might not have been correctly classified and recorded as a maternal death. Therefore, our finding of 32 maternal deaths could be an underestimation, which has been indicated in previous hospital studies [23,24].

One strategy researchers may consider using is the Rapid Ascertainment Process of Institutional Deaths, which was developed by the Initiative for Maternal Mortality Program to overcome institutional maternal death underestimations [23]. In summary, this strategy entails identifying all possible units/wards within the hospital where women of reproductive age (15-49 years) might receive care, reviewing death records of these women and assessing their pregnancy-related status.

### Data collection

#### Chart extraction

Because medical charts typically are multi-authored with each healthcare worker choosing what they think is relevant or important to record, the quality of documentation within and across medical charts vary [25]. For all the key elements in a medical record—history, physical findings, test results, etiology, exposures, severity scales and scores, discharge diagnoses, and outcomes—healthcare providers differ in how they observe, measure, interpret, and report their findings [26]. Therefore, the quality of the data extracted is dependent on the completeness and accuracy of what the healthcare providers actually document in the record [27].

When there is no written entry in the medical chart, this does not necessarily mean that care or treatment was not given [28]. Other possible explanations for missing information could be that the healthcare providers failed to elicit important information, failed to record information they obtained or care they gave, or that patients could not recall their symptoms and illnesses or they deliberately withheld pertinent information [27]. In the past there was an attempt to introduce standardized records so that essential information would always be recorded and known. However, because of limited funding from the University of North Carolina, and no financial support from the Ministry of Health to sustain the activity, standardized record keeping has not been completely institutionalized.

Regardless of the reason, if the data extractors could not find the relevant information in the chart, they searched among the supporting documents or solicited the information during the interviews. Information might have been missed, particularly if the information of interest was presented as a single word, or only reported once in the entire text [29]. In summary, the information documented within medical charts has been characterized in the literature and through our observations as being poorly maintained and marred with errors, omissions, and idiosyncrasies [26,30,31]. However, the data extractors were still able collect some valuable data and triangulate it with the other information to glean a better picture of what did and did not occur.

Using medical charts as a source of data is inevitable for such retrospective investigations. Therefore researchers must recognize medical charts’ inherent limitations and challenges. Researchers must adhere to methodological reporting standards such as those outlined by Worster et al [32] so that readers can determine the applicability, validity, and reliability of the results. Researchers should also take steps to add rigor to the data collection process, such as those outlined by Gregory and Radovinsky [33].

Missing charts are problematic at the research level and at the facility level, particularly with regard to the facility’s clinical governance. At the research level, missing data weaken the strength of argument that the data presented may have. This is the case when data are being used to show trends in causes of death, which may in turn be used to inform policy-makers for deciding which areas within a national healthcare plan one should invest resources in an attempt at (e.g., reducing maternal deaths). However, if the “take home messages” vary from case to case, with little focus on trends within the cohort, then the absence of medical charts simply reduces the number of anecdotes from which to learn. The strength of the anecdotes from existing cases is not reduced in their potential impact.

The implication of the missing charts at the facility level is dependent on whether there is a system in place for health information collection and storage in cases of maternal mortality occurring in the facility. There was no system in the facilities where the maternal death review study was conducted. Institutionalizing such a system is a matter of urgency if future maternal death reviews are expected to fulfil their function and provide an accurate representation of the case burden for the facility. In this scenario missing cases could be assumed to be of equally random assortment as those reported.

In instances where medical charts for maternal deaths cases are missing and a health information system for routinely collecting maternal death indicators exists, then the question must be asked, “Why does the existing system not work?” The system in place might have a poor design that could never function efficiently even with the best of intentions. Another possibility is that the design is good but the environment in the institution is not conducive to openness regarding the maternal death review process because of partiality of reviewers, and the fear of individuals being held accountable (and punished) rather than there being an interest in looking for system failure. In this type of environment, particular causes of death might be covered up through notes going missing. The implications of this speculation are serious and far reaching, not least from a legal perspective. A facility should have sufficient self-interest to at least address this speculation as a possibility. In this second scenario, the missing charts might be related to certain causes of death that call into question the competency, staffing and medical resources available within the facility. This would introduce a stronger bias if trends from existing data did not address the shortcomings of the care provided for those cases where charts were missing.

To avoid the issue of medical charts missing altogether, researchers may consider conducting a prospective study where he/she can keep a watchful eye over the transfer of the charts. Otherwise, rules about how missing charts and data will be handled should be devised prior to the commencement of the data collection process. During the data collection process researchers should determine the degree of missing data and charts and whether they are missing by random chance. The most effective method to determine the development of any problems from missing data or charts is to conduct a pilot study [34].

#### Facility-based interviews

We found that although hospital staff was generally helpful, they were extremely busy. This created a need for researchers to have a great deal of flexibility, reciprocity and time to develop relationships with the staff, discover established routines and practices and to conduct research in such a setting. In addition to reviewing schedule rosters, researchers may want to consider consulting an independent medical doctor to help determine whom to interview. If appropriate and feasible, at least one healthcare worker should be interviewed at each place where the woman in a given case was admitted.

Because of the ad hoc and almost exclusively negative or corrective nature of supervision [35], some of the participants were fearful to speak openly. Kongnyuy and van den Broek also reported that interviewees might have concealed information because of a fear of being blamed [4]. Therefore, it is important for researchers to effectively convey the purpose of such investigations, which is to learn from such tragedies to prevent them in the future and to not apportion blame. In cases where the death is controversial researchers should have more than one perspective from the same unit where the death occurred. Moreover, the data collection tool used should be piloted.

#### Community-based interviews

Consent was obtained from of all the participants, although true voluntary participation was questionable for the following reasons. First Malawian chiefs, similar to other African chiefs, play a critical role in the consent process [36-38]. Participants might have obliged because the chiefs were present. However, this might not have been the case as noted in a study conducted in rural Ghana [38]. In this previous study, individuals enrolled in research not because they feared disobeying community leaders, but because they believed the chief would not have granted permission for the study if he thought it was not in their best interest to join. However, in traditional, rural African communities people are considered as members of the society, rather than as autonomous individuals [39].

To address concerns about cultural obligation, eligible participants must always be told, especially in the presence of community leaders, that participation is voluntary and that they are free to withdraw or to not answer any question at any time if they choose. Researchers should always bear in mind these potential ethical issues while trying to accommodate local cultural norms in decision making.

Participants can be candid when the recorder is off as opposed to when it is on [40,41]. Negative viewpoints about care provided and about care being withheld were mentioned “off the record” by some of the participants (both healthcare workers and family members) in our study. However, because more than one family member and healthcare worker were interviewed for several cases, information that was off the record by one of them was mentioned as on the record by another. Therefore, off-the-record accounts had no bearing on the conclusions or recommendations made, which is why it is imperative for researchers to include several perspectives for a given case.

In general, it is critical that during the research planning/data collection form development phase experts who have lived and or worked in the area(s) of interest are consulted to gain insight and a better appreciation of the concepts of time, distance and cultural norms. Researchers should also not underestimate the time required to complete this task of locating and interviewing appropriate family and community members. Therefore, assistance from local authorities who are presumably able to locate participants more efficiently and effectively than an outside researcher should be solicited.

With regard to language barriers, researchers need to address the methodological issues surrounding language barriers between researchers and participants as systematically as possible. This can be achieved by adhering to Squire’s 14 methodological recommendations to maximize the trustworthiness of data obtained, analyzed, and reported with the assistance of an interpreter or translator [42].

### Reviewing cases and analyzing data

Given that autopsies are rarely performed in Malawi, the cause of death must be elucidated through a review of the patient’s notes. Details must be gleaned from interviews conducted with clinical staff and the patients’ relatives, or other appropriate persons. The quality of the chart review itself is dependent on the accuracy of the documented working diagnosis at the time of death. This in turn is dependent on the thoroughness of the patient’s “work up” after making contact with health professionals. In clinical practice, there is often disagreement among health professionals in interpreting histories, physical signs of disease, diagnostic tests, and other clinical information [27,43]. We also observed that clinicians varied in the detail and accuracy with which they recorded their observations.

### Causes of death and International Classification of Diseases

A differential diagnosis is a complex reasoning process that begins with the patient's individual illness history and culminates in a logical construct that can be categorized [44]. This process involves a large amount of information, including the patient’s lifelong and medical history, the patient’s report of their current medical problem, and the results of numerous examinations, procedures, and tests [45]. In addition to this information the clinician must possess a large amount of knowledge about health and disease as well as a degree of intuition [45,46]. Furthermore, medical demarcations, inclusion criteria and definitions are constructed by the current level of biomedical knowledge and consensus which also influence the differential diagnosis. If correct, the formulations of a differential diagnosis will lead to the appropriate treatment, which is important to patient and clinician.

Concurrently, the "cause of death" that is documented on a death certificate of a maternal death should reflect the state of pathology leading to the women's demise and not the mode with which the person met her death [47]. An example of this situation is cardiac arrest in a postpartum woman - where an event is described that if left untreated to a definite mode of death - the heart stops. However, this does not explain why the heart stopped in the first place which could be the end result of a cascade of events that eventually kills the woman. She may have suffered from malnutrition since childhood and be stunted and anemic, and also have intestinal worms that lead to worse chronic anemia. The anemia is made worse by three subsequent pregnancies within a short time period, and a birth that is complicated with a long second stage and postpartum hemorrhage, eventually lowering her hemoglobin to the point that heart function is hampered. The arrest could also be caused by massive intrapartum bleeding caused by an abruptio placenta that was not related to deprivation. A philosophical or political standpoint could come into play when deciding which interventions, at what point in the hierarchy of causality and when interventions are introduced with a high probability that they will reduce the number of deaths caused by a specific factor [48].

On reviewing the cases and the causes of death provided by the clinicians involved in the patients’ care, the following conclusion can be made: the less an investigation is performed prior to a patient’s demise, the more the “cause” of death stated in the notes reflects a patient’s mode of death as opposed to the pathological state causing a patient’s death. The more that the clinician’s stated cause of death reflects the patient’s mode of death (e.g., “hypoxia” – lack of oxygen), the greater the likelihood of drawing inferences in the conclusions.

The ICD-10 was used for classifying the causes of maternal death in the 32 cases that were reviewed. Although the causes of maternal death stated in the medical charts contained a degree of inference and a lack of confirmation through the positive identification of microbes or autopsies, the histories retold by clinicians and relatives (when available) offered valuable insight into the pathological processes affecting the patient.

For a direct cause of death, such as post partum hemorrhage, there is a specific code within the ICD-10 system for denoting this condition. However, where applicable and possible, for those with an indirect cause, one may have to provide a specific cause that is found outside of Chapter 15 relating to pregnancy, birth and puerperium (codes containing the prefix “O”), but that includes a code within Chapter 15 that best describes the organ system affected in the process leading up to the woman’s death. An example of this is the use of code O99.4 - *Diseases of the circulatory system complicating pregnancy, childbirth and the puerperium*. This code was applied in one of the cases in which the woman died as a result of heart failure secondary to severe anemia in pregnancy. Although the code may seem vague, and closer to a description of the woman’s mode of death, there are often few specific details that can be found because of a lack of a confirmed diagnosis in the woman’s notes. Nonetheless, by denoting a code from Chapter 15, no matter how vague, the routes by which women meet their death can be speculated in a broad and quantifiable sense.

Denoting a code from Chapter 15 has its usefulness in the setting of a continual audit of maternal deaths. If one notices that the number of maternal deaths denoted by code O98.6--Protozoal diseases complicating pregnancy, childbirth and the puerperium-- is on the rise then it is clear that interventions targeting parasitic disease in pregnancy are failing. In one geographical setting the culprit may be *Plasmodium falciparum* which is the parasite responsible for malaria. While in another geographical setting the culprit may be gastrointestinal worms which lead to malnourishment and severe anemia.

In many of the 32 cases more than one ICD-10 code applied. We did not assume that the codes would always describe causation and not just correlation. An example of this problem is when a patient suffers from AIDS, thereby contributing to her generally poor health status. As a consequence she is not able to withstand health insults well and could die from an insult, (e.g. aspiration pneumonia as a result of an anesthetic mishap, from which a patient not suffering from AIDS might not die). Therefore, AIDS in this case would not have been the direct cause of death. However, the direct cause is unclear because neither the anesthetic mishap, nor the resulting complication would have necessarily led to her death if she had not suffered from AIDS. Therefore, it is not always possible to be sure about causation or association. In sum, the codes given in the current review of cases represented the best possible guess in cases in which the information was primarily non-medically confirmed.

The OB/GYNs who analyzed the data were as susceptible to error as any clinician. Additionally their conclusions were based on their respective clinical judgments in the absence of reliable clinical or laboratory data to supplement the diagnostic procedure. In general, where disagreement in diagnosis occurred, it was usually an attempt at clarifying the most likely underlying cause, as opposed to a mode of death given the limitations of the details provided. In some cases, the reviewer’s cause of death was vaguer than that of the clinician’s. This is because the reviewer was not always convinced of the certainty of the suggested cause (e.g. “valvular heart disease”) which at times appeared inferred rather than confirmed, but concurred in the mode of the woman’s demise (e.g. “congestive cardiac failure”). Clinicians often want to have a diagnosis, and therefore, they choose one that is most likely [49]. The fact that an OB/GYN can make reasoned speculations as to what contributed to a maternal death is beneficial regardless of whether there is a disagreement with the diagnosis found in the patient’s file. Discrepancies are invitations to investigate the case further, perhaps divulging aspects to care that had not been examined.

For researchers, having a basic working knowledge of the ICD and its limitations are beneficial. To enhance the rigor of the classification process, researchers may consider involving at least two physicians who can independently review and classify the causes of maternal deaths in question. Alternatively, researchers may consider establishing a review team of experts who would review the deaths as a group and come to a consensus on the causes of death.

### Recommendations and evaluation

As stated earlier, recommendations were formulated and presented without any problems to senior management and staff at both hospitals where the study was conducted. The issue of recommendations lies in the question of whether the recommendations will be acted upon and subsequently evaluated. Previous studies have shown that recommendations resulting from the audit sessions were poorly implemented because of a lack of human and material resources and insufficient staff and senior management commitment [13,14]. Conversely, in other studies where recommendations were implemented and evaluated, reductions in maternal mortality, the case fatality rate [50,51], the incidence of uterine rupture, obstetric hemorrhage and the number of women with severe maternal complications were observed [52,53].

For researchers, it is critical to allot more time to share findings with all stakeholders, including community members, solicit feedback and involve them in the formulation of recommendations. Researchers may also consider developing an evaluation plan or at least identify variables to be included in a monitoring or evaluation plan.

## Conclusions

The facility-based maternal death review approach is not novel, and neither is the five-step maternal mortality surveillance cycle that underpins it. The approach and steps used in the current study were useful in ascertaining in-depth information on clinical, sociocultural, and other contributory factors of the maternal deaths reviewed, but were not without some challenges. Readers seldom gain an in-depth view of how complicated this process can be. In fact, in published research articles, books and reports, the data collection process of the facility-based maternal death review approach tends to be presented in a brief, sterile and sometimes perfunctory manner.

This paper attempted to bring potential methodological challenges to the forefront and provide some recommendations to help resolve them. Anticipating these challenges and developing strategies to minimize any negative effects offer the best chance for successfully completing a facility-based maternal death review despite the uncertainties of the environment in which it is conducted.

## Endnote

^a^The government of Malawi began training TBAs in the late 1970s. At the end of the 20^th^ century training was discontinued. In 2007, an official ban on TBAs by the Government of Malawi was in effect. As such, TBAs were required to refer all of their patients to the nearest health facility. Chiefs went as far as penalizing couples who went to TBAs (who didn’t deliver in the health facility) by charging/fining them a goat. In 2010 the late president Mutharika lifted the ban on TBAs. He stated that TBAs shouldn’t be abandoned but instead be trained and supported as a way of dealing with Malawi’s high maternal mortality ratio. In 2012 the ban was back in effect by President Joyce Banda. She demanded that TBAs must stop delivering babies, and because of their experience they could serve as a referral point.

## Competing interests

The authors declare that they have no competing interests.

## Authors’ contributions

VCT was responsible for the study conception and design, and undertook part of the data collection. VCT and JS were responsible for data analysis. VCT was responsible for drafting the manuscript. All authors made critical revisions of the paper, and all authors read and approved the final manuscript.

## Authors’ information

VCT has a PhD in international community health. She is a lecturer and has worked in Lilongwe, Malawi since 2005 in the areas of HIV-related stigmatization and maternal health. TM is the former head of the Department of Obstetrics and Gynecology at the studied hospital for the current study. TM previously worked as an OB/GYB for six years and as a general practitioner for four years in a hospital in northern Namibia. TM also holds a Master’s degree in international human rights law from Oxford University and is currently an Associate Professor and Head of Department of Obstetrics and Gynaecology at the University of Namibia, School of Medicine. JS is an OB/GYN and professor with research training in epidemiology and medical anthropology. JS teaches international health in postgraduate programs and supervises students majoring in reproductive health from Norway and several African countries. JS has worked in seven African countries, including Malawi, for over two decades. AM holds a PhD in Nursing from Edith Cowan University in Australia, has worked for the University of Malawi, Kamuzu College of Nursing (KCN) since 1989 and has been the Principal of KCN since 2008. AM is also the current president of the Association of Malawian Midwives and the president of Tau Lambda-at-Large (Africa Chapter) of the Sigma Theta Tau International.

## Pre-publication history

The pre-publication history for this paper can be accessed here:

http://www.biomedcentral.com/1471-2288/14/29/prepub
